# Blueprint for a High-Performance Biomaterial: Full-Length Spider Dragline Silk Genes

**DOI:** 10.1371/journal.pone.0000514

**Published:** 2007-06-13

**Authors:** Nadia A. Ayoub, Jessica E. Garb, Robin M. Tinghitella, Matthew A. Collin, Cheryl Y. Hayashi

**Affiliations:** Department of Biology, University of California Riverside, Riverside, California, United States of America; American Museum of Natural History, United States of America

## Abstract

Spider dragline (major ampullate) silk outperforms virtually all other natural and manmade materials in terms of tensile strength and toughness. For this reason, the mass-production of artificial spider silks through transgenic technologies has been a major goal of biomimetics research. Although all known arthropod silk proteins are extremely large (>200 kiloDaltons), recombinant spider silks have been designed from short and incomplete cDNAs, the only available sequences. Here we describe the first full-length spider silk gene sequences and their flanking regions. These genes encode the MaSp1 and MaSp2 proteins that compose the black widow's high-performance dragline silk. Each gene includes a single enormous exon (>9000 base pairs) that translates into a highly repetitive polypeptide. Patterns of variation among sequence repeats at the amino acid and nucleotide levels indicate that the interaction of selection, intergenic recombination, and intragenic recombination governs the evolution of these highly unusual, modular proteins. Phylogenetic footprinting revealed putative regulatory elements in non-coding flanking sequences. Conservation of both upstream and downstream flanking sequences was especially striking between the two paralogous black widow major ampullate silk genes. Because these genes are co-expressed within the same silk gland, there may have been selection for similarity in regulatory regions. Our new data provide complete templates for synthesis of recombinant silk proteins that significantly improve the degree to which artificial silks mimic natural spider dragline fibers.

## Introduction

Spider silks have received much economic and biomedical attention because of their outstanding mechanical properties [e.g. 1]–[3]. For example, the dragline silk of araneoids (ecribellate orb-weaving spiders and their relatives) displays both high tensile strength and extensibility, making it tougher than nearly all other natural or synthetic materials [4]–[6]. Spider silks are primarily composed of proteins that are synthesized in specialized abdominal glands. An individual orb-weaving spider spins up to five different types of silk fibers, each serving critical ecological functions, including prey capture, shelter, predator avoidance, egg protection, and dispersal [7]–[8]. Each distinct fiber is made from one or two unique types of silk structural proteins (fibroins), almost all of which are encoded by members of a single gene family [9]–[12]. Thus, the spectacular diversity of spider silk proteins evolved through successive rounds of gene duplication and divergence.

Spider fibroins have very high molecular weights, estimated at 200–350 kiloDaltons [13] with transcript sizes of approximately 10,000 base pairs (bp) or larger. Such considerable size is conserved over a diverse range of spider species and fibroin types [13]–[16]. Partial-length complementary DNA (cDNA) sequences indicate that silk proteins are highly modular; each polypeptide is primarily composed of an uninterrupted block of repetitive sequence that is flanked on both sides by ∼100 amino acids (aa) of non-repetitive amino- (N-) and carboxy- (C-) termini. The sequence attributes of the repetitive region vary according to silk protein type, with some fibroins containing short, simple repeat units, and others composed of longer, more complex repeats [10], [14]. Because of the difficulty associated with cloning long stretches of repetitive DNA, only two full-length cDNA silk sequences have been characterized [16]. These cDNAs encode the silk proteins that form the egg case fibers of the orb-weaving spider, *Argiope bruennichi*. Egg case fibers, however, have substantially lower tensile strength and toughness than dragline silk [8], [16]. Complete gene sequences are still unknown for any spider silk.

Because of its extremely high tensile strength and toughness, dragline (major ampullate) silk has received the most attention of the spider silks. This silk is composed of two types of fibroins, MaSp1 [17] and MaSp2 [18]. The genes encoding these proteins are co-expressed in the major ampullate silk glands, and both proteins are found throughout the fiber [15], [19]. Short glycine-rich regions (GGX, where X represents a subset of aa) followed by a stretch of multiple alanines (poly-A) characterize both proteins. The ubiquitous poly-A stretches are hypothesized to form hydrophobic crystalline domains that are responsible for the high tensile strength of the fiber [20]–[24]. In contrast, the glycine-rich regions are hydrophilic with runs of the peptide motif GGX conforming to a 3_1_-helix [21], [25]. While poly-A and GGX motifs describe almost all of MaSp1, MaSp2 also has a large proportion of GPG motifs [18]. These proline-containing repeats likely form type II beta-turns, and such kinks in part explain the reversible extensibility of dragline fiber [13], [19], [26]–[27].

Much of the applied research on spider silks has focused on mass-producing silk fibers for industrial use [e.g. 28]. However, unlike domesticated silkworm caterpillars, spiders cannot be readily farmed for silk because they are predatory and cannibalistic. Instead, researchers have created biomimetic silks through transgenic technologies using partial-length silk cDNA sequences [e.g. 29]–[32]. These manmade silks have, thus far, fallen short of native dragline silk in both sequence and mechanical properties [e.g. 30], [33]–[35]. All known arthropod fibroins are extremely large, including the convergently evolved heavy-chain fibroin of lepidopterans (∼370 kiloDaltons [36]). Such evolutionary convergence among distantly related silk spinning species suggests that large size is a critical molecular feature for silk fiber mechanical performance. For example, larger fibroins possess more repeat units (such as poly-A motifs that crosslink to form crystalline domains) than shorter fibroins, thereby increasing the number of interactions among monomers. However, large size is probably not the only key functional attribute of spider fibroins. The evolutionarily conserved C-termini of MaSp1 and MaSp2 aid in conversion of the liquid silk dope into a solid fiber [37], and facilitate assembly of the fiber's characteristic crystalline domains [35]. The few known N-termini are even more conserved than the C-termini [38]–[39], which suggests that N-termini are also necessary for proper fiber assembly and may influence the mechanical properties of spider silk. Thus, determining the entire coding sequences for MaSp1 and MaSp2 is a key step in the generation of recombinant silks that closely mimic natural spider dragline silk.

Full-length silk sequences are also crucial for understanding the molecular evolutionary dynamics of long, repetitive genes. To date, it has been assumed that the modular organization of fibroins is maintained throughout the entirety of the amino acid sequence. Yet, some molecular evidence is consistent with past recombination events between *MaSp1* and *MaSp2* [10], [12], [40], and thus repeat units may have transferred from one gene to the other. Moreover, there is sparse information about the exon-intron structure of spider silk genes. *Flag* (the gene encoding the capture spiral filament) from *Nephila clavipes* [41] and *MaSp2* from *Argiope trifasciata* [38] both show highly repetitive exon-intron gene organizations in which sequential introns within a gene have nearly identical nucleotide sequences. The presence of iterated introns and exons is evidence that intragenic recombination can swiftly homogenize (eliminate or spread new variants throughout) the sequence of an entire silk gene. It is unknown if this atypical gene architecture is a common feature of spider silk genes.

We have constructed a genomic library for the black widow spider, *Latrodectus hesperus* (Theridiidae), in order to identify full-length silk genes and their associated regulatory regions. Black widows, notorious for their neurotoxic venom, are members of the Araneoidea, a superfamily of orb-weaving spiders and their close relatives. Black widows are descended from orb-web weaving ancestors, but they build three-dimensional cobwebs rather than the symmetrical, wagon-wheel shaped orb-web [42]. Despite this difference in web architecture, the breaking strength and extensibility of *Latrodectus* dragline silk are equal to or higher than those of true orb-weaving spiders [43]–[45]. Here we report complete gene sequences of *MaSp1* and *MaSp2* as well as adjacent non-coding regions. We document the existence of higher-order repeat units that range from ∼70 to over 2,000 bp, and show that the repetitive sequences of *MaSp1* are more homogenized than those of *MaSp2*. We also demonstrate marked evolutionary conservation of N-terminal and upstream non-coding regions between paralogs within a species and across orthologs from divergent species. Based on these multi-gene comparisons, we identify putative regulatory sequences that may be involved in co-expression of the two major ampullate silk genes. Collectively, our data provide the first templates for complete recombinant major ampullate fibroins and illustrate the dramatic effects of intragenic and intergenic recombination in the evolution of these extraordinarily modular genes.

## Results

### Large, single exon gene organizations

**Figure 1:**
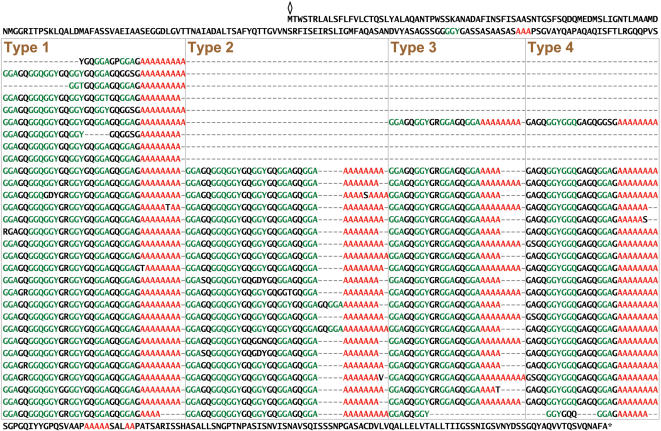
Complete amino acid sequence for *L. hesperus* major ampullate spidroin 1 (MaSp1). The sequence is read from left to right and then top to bottom. The diamond marks the start position and the asterisk denotes the stop position. The protein is dominated by poly-A (red) and GGX (green) motifs. The majority of the sequence can be categorized into four types of ensemble repeat units. Repeats of each type are aligned within a box. Gaps (-) have been inserted in order to align repeat units within a type.

**Figure 2:**
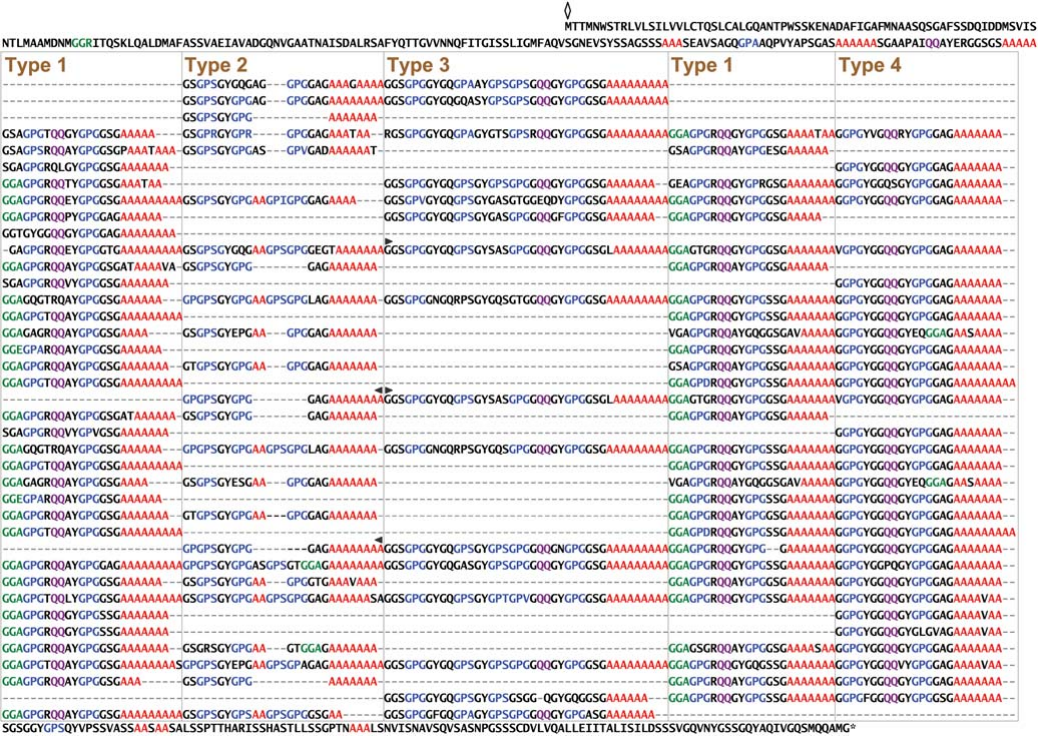
Complete amino acid sequence for *L. hesperus* major ampullate spidroin 2 (MaSp2). The sequence is read from left to right and then top to bottom. Start position, stop position, and alignment gaps are indicated as for MaSp1 (Figure 1). MaSp2 is characterized by poly-A (red), GGX (green), GPX (blue), and QQ (purple) motifs. There are four types of ensemble repeats. Repeats of each type are aligned within a box, except for Type 1, which is separated into two boxes because it is approximately twice as prevalent as any other repeat type. Right and left pointing arrows mark beginnings and ends of two near-perfect repeats of 778 aa.

We sequenced two fosmid clones each containing ∼37,000 bp of the black widow genome. One clone (GenBank accession EF595246) encompassed the complete coding sequence for the dragline silk gene *MaSp1* as well as 9,928 bp upstream of its start codon and 14,728 bp downstream of its stop codon. The *MaSp1* gene is composed of a single exon with 9,390 bp encoding 3,129 aa (Figure 1). The second clone (EF595245) includes the entire coding sequence for *MaSp2* plus 17,205 bp of upstream and 8,546 bp of downstream flanking sequence. Like *MaSp1*, the *MaSp2* gene contains one enormous exon with 11,340 bp encoding 3,779 aa (Figure 2). Both *MaSp1* and *MaSp2* genes contain sequences that match partial-length cDNAs from *L. hesperus* silk gland expression libraries [40], [43], indicating that these genes are transcribed. The C-terminal coding region (∼300 bp) of the *MaSp1* gene is 97% identical to the corresponding 3′ partial *MaSp1* cDNA clones (AY953074, DQ409057) and the N-terminal coding region (∼450 bp) is 99.8% identical to our 5′ partial cDNA clone (EF595247). Both the C-terminal coding region and the 3′ untranslated region (UTR) of the *MaSp2* gene share 99% sequence identity with 3′ partial *MaSp2* cDNA clones (AY953075, DQ409058). Similarly, the N-terminal coding regions of the *MaSp2* gene and our 5′ partial cDNA (EF595248) are 95.5% identical.

### Extreme sequence modularity

Glycine and alanine are by far the most abundant amino acids in our predicted *L. hesperus* MaSp1 and MaSp2 fibroins. These two amino acids constitute greater than 64% of both sequences, followed by glutamine in MaSp1 and proline in MaSp2 (Table 1). These values closely match published amino acid compositions of major ampullate silk from black widows [46] and other araneoid spiders [47]–[48], further confirming that our genes encode the two dominant protein components of major ampullate silk. Because the first two codon positions for alanine, glycine, and proline are guanine or cytosine, the base compositions of these genes are guanine/cytosine-rich (*MaSp1*–61%; *MaSp2*–59%). However, overall base compositions are not highly skewed because the third positions for these codons in the *L. hesperus MaSp1* and *MaSp2* are extremely biased towards adenine and also strongly biased, but less dramatically, towards thymine (86% of *MaSp1*, 91% of *MaSp2* glycine, alanine, and proline codons end with adenine or thymine; Table 1).

**Table 1:** Amino acid content and codon usage for the most common amino acids of black widow MaSp1 and MaSp2.

Amino Acid	Codon	MaSp1		MaSp2	
		% aa	% codon	% aa	% codon
Glycine	GGA	42.3	54	33.5	65
	GGT		38		30
	GGC		7		4
	GGG		1		1
Alanine	GCA	32.7	59	31.1	66
	GCT		18		18
	GCC		17		7
	GCG		6		9
Glutamine	CAA	11.3	98	6.9	97
	CAG		2		3
Proline	CCA	0.4	69	8.6	64
	CCT		23		33
	CCC		8		1
	CCG		0		2

The repetitive region of the *L. hesperus MaSp1* translation is dominated by amino acid sequence motifs commonly found in MaSp1 of other spider species: GGX (X = A, Q, or Y), GX (X = Q, A, or R), and poly-A (4–10 consecutive alanines, mean number = 7.7) [10], [17], [49]. These motifs are organized into four types of ensemble (higher order) repeat units, with each ensemble consisting of a glycine-rich region followed by a poly-A region (Figure 1). Starting at residue 542, the different ensemble types are tandemly arrayed in a consistent pattern, and this aggregate of four ensembles is iterated 20 times with near perfect fidelity. Pairwise amino acid differences between aggregates are extremely low, ranging from 0.0 to 4.3% and averaging 1.9%. This remarkable sequence homogeneity is also maintained at the nucleotide level with average uncorrected pairwise differences of only 2.5% (range = 0.3–6.3%).

The repetitive region of the *L. hesperus* MaSp2 amino acid sequence is characterized by a larger suite of motifs than MaSp1. The common MaSp2 motifs include GPX (X = G or S), QQ, GGX (X is usually A), GSG, and poly-A (3–9 consecutive alanines, mean number = 6.7). Similar to MaSp1, these motifs are organized into four types of ensemble repeat units that each contain one poly-A motif (Figure 2). However, the four types of MaSp2 ensembles are more variable than those of MaSp1, with pairwise amino acid differences between ensembles of the same type as high as 36% (Table 2). In addition, the MaSp2 ensemble types are not always strung together in the same order and do not form clearly discernible higher-level aggregates (Figure 2). Nevertheless, there is a pair of 778 amino acid long tandem repeats that differ by a scant five aa (Figure 2). The 2,334 nucleotides encoding each repeat vary at only six positions (>99.7% identity).

**Table 2:** Prevalence (#) and average pairwise amino acid differences between MaSp2 ensemble repeats of the same type.

Ensemble Type*	#	Average % aa difference (min-max)
1	62	11.8 (0.0–36.0)
2	24	11.7 (0.0–28.0)
3	16	11.4 (0.0–22.0)
4	30	5.6 (0.0–20.8)

* Ensemble repeat types shown in Figure 2.

**Figure 3:**
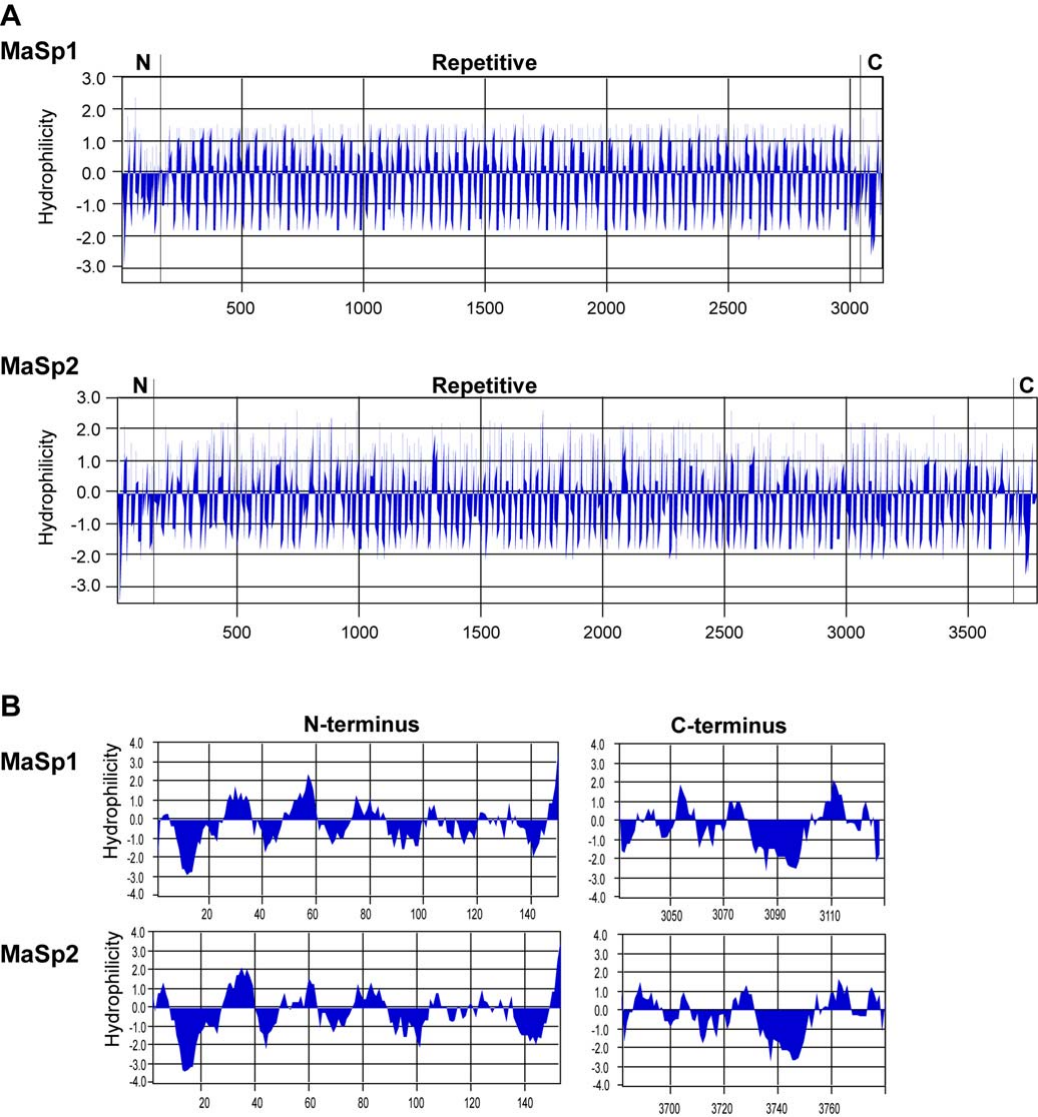
Kyte and Doolittle [50] hydrophilicity plots for *L. hesperus* MaSp1 and MaSp2. Scan window size = 7. Negative values indicate hydrophobicity. (A) Complete proteins. (B) Non-repetitive terminal regions.

Using the method of Kyte and Doolittle [50], we predicted the hydrophilicity of *L. hesperus* MaSp1 and MaSp2. Both fibroins show regions of hydrophobicity (corresponding to the poly-A motifs) and hydrophilicity (corresponding to the glycine-rich regions) that alternate throughout the entire repetitive portions of the two proteins (Figure 3A). Both MaSp1 and MaSp2 repetitive regions are slightly hydrophilic when averaged across all residues (MaSp1 average = 0.13 on the Kyte-Doolittle scale; MaSp2 average = 0.14) but MaSp2 displays higher amplitude of hydrophilicity (MaSp1 max = 2.0; MaSp2 max = 2.6) (Figure 3A). The N- and C-terminal domains show a similar pattern of alternating hydrophobicity and hydrophilicity but are generally more hydrophobic than the repetitive regions (average hydrophilicity across residues: MaSp1 N-terminus = -0.29, C-terminus = −0.44; MaSp2 N-terminus = −0.34; C-terminus = −0.31; negative values indicate degree of hydrophobicity). The most hydrophobic region of both fibroins is found at the beginning of the N-terminus (Figure 3B).

### Congruence between silk N- and C-termini evolutionary relationships

**Figure 4:**
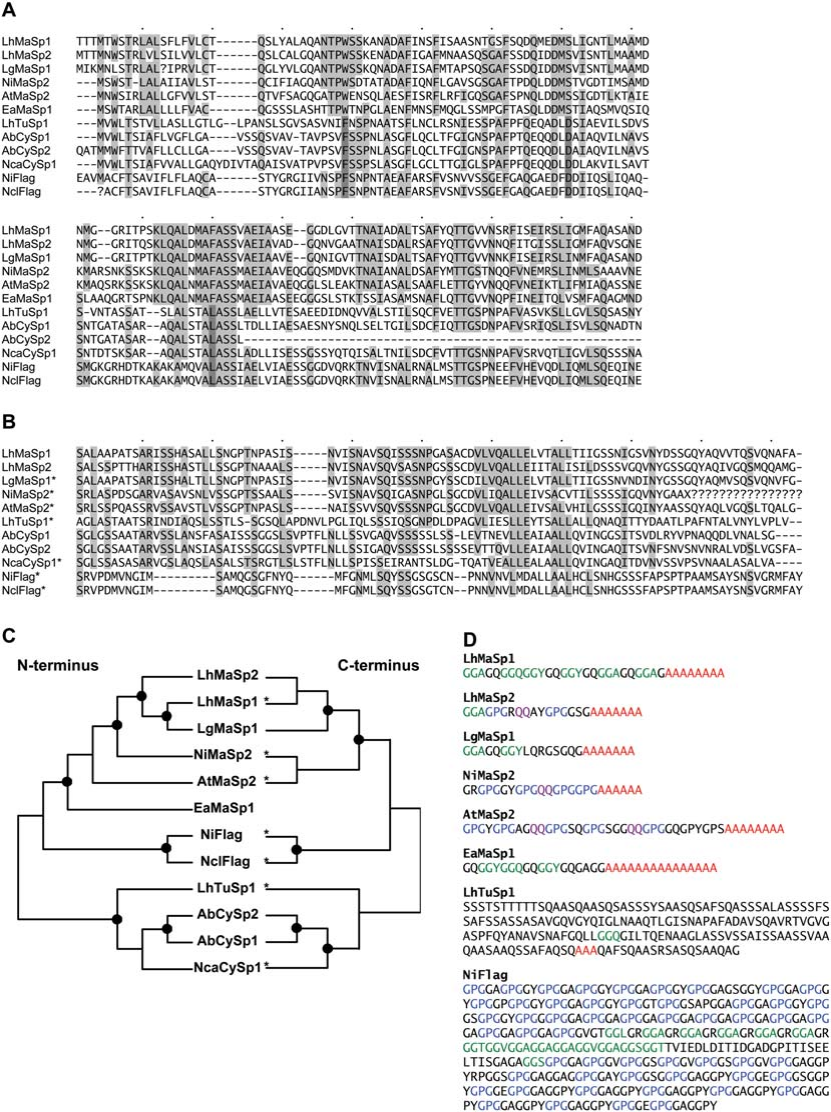
Comparison of N-termini, C-termini and repeat units of spider silk proteins. (A) Alignment of published N-terminal amino acid sequences. Amino acids shared by ≥50% of proteins are highlighted in grey. Gaps are represented by dashes and missing characters by question marks. (B) Alignment of corresponding C-terminal amino acid sequences. Taxa with an asterisk result from partial sequencing and are presumed to belong to the same locus as the N-terminal sequences. (C) MP trees of N and C-terminal encoding sequences treating gaps as a fifth state and employing midpoint rooting. Left tree length = 1449 (N-terminus); Right tree length = 838 (C-terminus). Dots represent nodes with >75% bootstrap support in all MP and ML analyses and >95% Bayesian posterior probability. (D) Exemplar repeat units for each of the major ampullate fibroins and representative TuSp1 and Flag repeats. Amino acid motifs are colored as in Figure 2. Abbreviations: LhMaSp2, *Latrodectus hesperus MaSp2* (EF595245); LhMaSp1, *L. hesperus MaSp1* (EF595246); LgMaSp1, *L. geometricus MaSp1* (5′ sequence: DQ059133S1, 3′ sequence: DQ059133S2); NiMaSp2, *Nephila inaurata madagascariensis MaSp2* (5′ sequence: DQ059135, 3′ sequence: AF350278); AtMaSp2, *Argiope trifasciata MaSp2* (5′ sequence: DQ059136, 3′ sequence: AF350266); EaMaSp1; *Euprosthenops australis MaSp1* (AM259067); LhTuSp1, *L. hesperus TuSp1* (5′ sequence: DQ379383, 3′ sequence: AY953070); AbCySp1, *A. bruennichi CySp1* AB242144; AbCySp2, *A. bruennichi CySp2* (AB242145); NcaCySp1, *N. clavata CySp1* (5′ sequence: AB218974, 3′ sequence: AB218973); NiFlag, *N. i. madagascariensis Flag* (5′ sequence: AF218623S1, 3′ sequence: AF218623S2); NclFlag, *N. clavipes Flag* (5′ sequence: AF027972, 3′ sequence: AF027973).

We aligned the N-terminal regions of *L. hesperus* MaSp1 and MaSp2 with N-termini from other spider fibroins (Figure 4A). These proteins are constituents of three spider silk fiber types: the dragline silk composed of MaSp1 and MaSp2, the capture spiral filament of flagelliform silk protein (Flag), and the eggcase fibers produced from tubuliform (also called cylindrical) gland proteins (TuSp1, CySp1 and CySp2). For each of these N-termini, we also aligned the corresponding C-termini, if available (Figure 4B). However, only in the case of the *L. hesperus MaSp1* and *MaSp2* that we report here, and the full-length *CySp1* and *CySp2* cDNAs from *Argiope bruennichi*, is it certain that the N- and C-termini coding regions belong to the same gene. All others were partial 5′ or 3′ sequences that were assumed to represent the ends of the same gene.

We assessed evolutionary relationships among the N-termini and C-termini encoding sequences using maximum likelihood (ML), maximum parsimony (MP), and Bayesian phylogenetic methods. All methods produced similar relationships among N-terminal sequences (Figure 4C). A clade of eggcase silks (TuSp1, CySp1 and CySp2) was always well-supported. A major ampullate silk clade (MaSp1 and MaSp2) was consistently recovered with greater than 90% bootstrap support and 100% posterior probability. Despite the distinct differences between the repetitive portions of MaSp1 and MaSp2 (Figures 1, 2, 4D), all N-termini analyses strongly supported a grouping of *Latrodectus* MaSp1 and MaSp2, rather than a multi-species MaSp1 clade that is distinct from a MaSp2 clade. Within *Latrodectus*, however, *L. hesperus* MaSp1 grouped with *L. geometricus* MaSp1.

Relationships among the corresponding C-terminal encoding sequences typically mirrored those of the N-terminal encoding sequences (Figure 4C). However, placement of the *L. hesperus* TuSp1 C-terminus was unstable. Depending on the type of analysis, it grouped with Flag, CySp1 and CySp2, or MaSp1 and MaSp2, but always with low support (less than 75% bootstrap support or 95% posterior probability). A MaSp1 and MaSp2 C-terminal clade was consistently recovered with high support values, and nested within it, a *Latrodectus* MaSp1 and MaSp2 sub-clade. The only difference among analyses was that *L. hesperus* MaSp1 grouped with either *L. geometricus* MaSp1 (ML and Bayesian trees) or *L. hesperus* MaSp2 (in the MP trees).

### Multi-species comparisons identify conserved non-coding sequences

Phylogenetic footprinting is a powerful approach for discovering putative gene regulatory regions. This method generally relies on alignments of orthologous, non-coding sequences from multiple species [51]. The presence of conserved non-coding nucleotide stretches implies that a region is under selective constraint and therefore is likely to perform an important function. A similar approach can be applied to the non-coding sequences of co-regulated genes [52]. We thus compared flanking sequences of *L. hesperus MaSp1* and *MaSp2*, paralogous genes which are simultaneously expressed [15], [19]. We also analyzed these sequences with available flanking sequences of *MaSp1* and *MaSp2* from other spider species. Because the *L. hesperus MaSp2* clone contained another open reading frame (ORF) 2,611 bp upstream of the *MaSp2* start codon, we limited comparisons to ∼2,500 bp of upstream sequence. Using MultiPipMaker [53], we identified regions that could be reliably aligned among *L. hesperus MaSp1* and *MaSp2, L. geometricus MaSp1* (5′: DQ059133S1, 3′: DQ059133S2), *Argiope trifasciata MaSp2* (DQ059136), and *Nephila inaurata madagascariensis MaSp2* (DQ059135; only 700 bp upstream sequence available). Downstream genomic sequences were not available for *Argiope* and *Nephila MaSp2*. MultiPipMaker generates local alignments using the BLASTZ algorithm and only produces an alignment if identity among sequences exceeds a threshold, below which alignments are considered random [Bibr pone.0000514-Schwartz2] [54−55]. Margulies et al. [56] argued that pairwise alignments are unreliable for detecting regulatory elements. Thus, we focused on conserved regions found in at least three sequences. When attempting to align only upstream non-coding sequence, MultiPipMaker produced alignments among *Latrodectus* sequences but not between *Latrodectus* and *Argiope* or *Nephila*. When the coding sequences were included as an anchor, a span of ∼90 bp directly upstream of the start codon could be aligned among all 5 genes. This region included the conserved motif CACG and the TATA box, which were also identified by Motriuk-Smith et al. [38]. While the TATA box is thought to guide RNA polymerase II to the transcription initiation site in many eukaryotic genes, the motif CACG represents a potentially novel regulatory element for spider silk genes. Approximately 150 bp of sequence upstream from the start codon could be aligned among the three *Latrodectus* genes and ∼300 bp upstream sequence between *L. hesperus MaSp1* and *MaSp2*. Additionally, ∼180 bp of sequence downstream of the stop codon could be aligned among all three *Latrodectus* genes.

We further investigated the regions of similarity identified among the *Latrodectus* non-coding sequences by creating global alignments of the ∼300 bp region upstream of the start codon and of the ∼180 bp segment downstream of the stop codon. In addition to the CACG motif and TATA box found among all sequences examined, the three *Latrodectus* upstream sequences share a 15 bp motif found ∼110 bp upstream of the start codon that has only 2 variable positions. When scanned against the TRANSFAC database [57], this conserved region perfectly matches a 6 bp binding site for the Achaete-Scute family of transcription factors.

**Figure 5:**
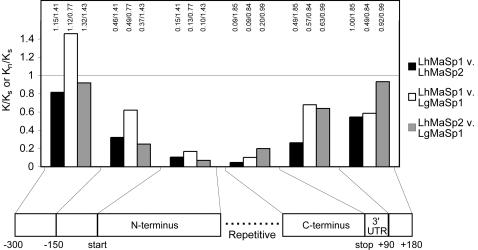
K/K_s_ or K_n_/K_s_ for flanking and terminal regions of *Latrodectus* major ampullate silk genes. K_s(N-terminus)_ is the denominator for upstream ratios; K_s(C-terminus)_ is the denominator for downstream ratios. Actual K values shown above bars. Gene abbreviations are the same as for Figure 4.

We also compared nucleotide substitution rates for various regions of the *Latrodectus* sequences (Figure 5). To detect selection on protein coding sequences, we calculated the ratio of the number of nonsynonymous substitutions per nonsynonymous site (K_n_) to the number of synonymous substitutions per synonymous site (K_s_) [58]. As expected for evolutionarily conserved proteins, we found K_n_/K_s_ was very low, ranging from 0.05 to 0.20 for *Latrodectus MaSp1* and *MaSp2* terminal coding regions, suggesting strong purifying selection (Figure 5). We applied an analogous approach (as in Wong&Nielsen [59]) to estimate selective pressures in non-coding sequences by calculating the ratio of the number of substitutions per site (K) to K_s_ for the adjacent coding sequence. We found K_(150 bp upstream)_/K_s(N-terminus)_ ranged from 0.26 to 0.63, which is higher than for coding sequence but still considerably less than 1. In contrast, K_(300−150 bp upstream)_/K_s(N-terminus)_ ranged from 0.82 to 1.45 (Figure 5), suggesting that the 150 bp directly upstream of coding sequence are under selective constraints while regions farther upstream are not. We also found K_(3′ UTR)_/K_s(C-terminus)_ = 0.27 for *L. hesperus MaSp1* and *MaSp2*, consistent with strong purifying selection on the 3′ UTR.

### Global comparisons of genomic clones

**Figure 6:**
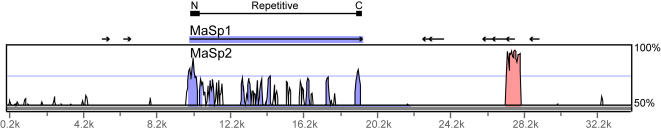
Global AVID alignment of *L. hesperus* genomic clones containing *MaSp1* and *MaSp2* visualized with VISTA. The *MaSp1*-containing clone was used as the reference sequence. Peak height corresponds to level of identity between the two clones. Regions exceeding 70% conservation over a window length of 100 bp are colored (blue for exons, red for non-coding sequence). The red peak corresponds to a putative transposable element found in both clones. Arrows mark open reading frames (ORFs) on the clone containing *MaSp1*.

We compared the entire clones containing *MaSp1* (34,046 bp) and *MaSp2* (37,092 bp) using MultiPipMaker and the global alignment program AVID [60]. We also compared the flanking sequences of the genes using BLASTN [61] to search for repetitive elements in the *L. hesperus* genome. As expected, the N- and C-terminal coding regions are significantly conserved between the two genes (Figure 6). Within the genes themselves, there are also multiple regions of significant similarity at the DNA level. These regions correspond to the poly-A, GG, GGXG and GQ motifs found in both proteins. Additionally, there were numerous significant matches between regions of non-silk-protein-coding sequence. Each of these regions, when translated, was similar to transposable elements in the NCBI nr protein database (based on BLASTX [61] scores: E<e^−10^). Most notably, there is a significantly conserved region spanning ∼700 bp that is found ∼10,000 bp downstream of the *MaSp1* and *MaSp2* ORFs (Figure 6). The translated sequence of this region from the *MaSp2* clone significantly matched TCb1-transposase. The translated sequence from the *MaSp1* clone significantly matched gag-pol polyprotein, which contains a retrotransposon. Although both clones contain ORFs in this region, they do not encode full-length proteins. Thus, these genomic regions appear to be inactive transposable elements.

## Discussion

### Gene structure

Black widow dragline silk is an exceptionally tough biomaterial, even compared to the high-performance draglines spun by other spiders [43], [45]. Here we report the complete gene sequences for the MaSp1 and MaSp2 fibroins that form this silk. We found that both genes lack introns and thus *MaSp1* and *MaSp2* each possess only one enormous exon containing either 9,390 bp (*MaSp1*) or 11,340 bp (*MaSp2*) of coding sequence. No other full-length spider silk genes have been characterized, but the few known partial-length gene sequences fit into two categories of exon-intron structure. First, based on *L. geometricus MaSp1* and *Nephila MaSp2* fragments [38], and the full-length genes described here, some silk genes are composed of single exons. Second, *Nephila Flag* and *Argiope MaSp2* have introns that are peculiar because successive introns within the same gene are nearly identical in sequence [38], [41]. Thus, all known spider silk genes have unusual architectures.

In eukaryotes, proteins encoded by single exons are rare and strongly biased towards sizes much smaller (<1,000 aa, [62]–[63]) than the spider silk proteins (>3,000 aa). Intronless genes may reflect one type of gene duplication process that led to the diversification of the spider silk gene family; retroposition of mRNA transcripts (inherently intronless) into the genome can give rise to functional gene duplicates [64]. Alternatively, intronless genes may be selectively favored. Intron length is negatively correlated with expression level [65]–[67] and major ampullate silk genes must be highly expressed throughout the lifetime of a spider. However, once an intron invades a silk gene, the intron can be rapidly propagated throughout the gene due to unequal crossing over, which appears to be common in silk genes (see Figure 2, [40]–[41], [49]).

MaSp1 and MaSp2 are almost entirely composed of a small suite of amino acid sequence motifs, such as GGX and poly-A, which are repeated many times throughout both fibroins (Figures 1, 2). In each fibroin, the frequency and arrangement of these motifs occur in four distinct types of repeat units, termed ensemble repeats. Although the ensemble repeats of both MaSp1 and MaSp2 are similar in length (∼30 aa) and composition (glycine-rich regions interspersed with alanine-rich regions), no repeat units from one protein are found in the other (Figures 1, 2). These results confirm that distinct genes encode each silk protein [e.g. 9], [17]–[18], rather than posttranscriptional processing of a single gene leading to silk protein diversity as previously suggested by Craig et al. [68].

Both *L. hesperus* MaSp1 and MaSp2 have glycine and alanine-rich motifs that occur in ensemble repeats, but the fibroins differ in their higher-level repeat organization (repetitiveness) and similarity of repeat copies (homogenization). In MaSp1, the four types of ensemble repeats are strung together to form an ∼120 amino acid long, higher-level repeat unit. This large aggregate is tandemly arrayed twenty times and the iterations share high identity at both the amino acid and nucleotide level (98.1% and 97.5% mean pairwise identity, respectively). In contrast, MaSp2 does not have clearly discernible higher-level repeats and has more sequence and length variation among its ensemble repeats than in MaSp1 (Figure 2, Table 2). MaSp2, however, has a tandem repetition of 778 aa that is >99.7% identical over the 2,334 encoding nucleotides (Figure 2). The modular architectures of MaSp1 and MaSp2 likely reflect concerted evolution within a single gene, as has been implicated in maintaining similarity among Flag (∼440 aa) ensemble repeats and the long repeats of TuSp1 (∼200 aa) and AcSp1 (aciniform silk; 200 aa [14]).

Modular architecture is also hypothesized to facilitate replication slippage in silk genes that have tandem arrays of codons for simple amino acid sequence motifs (e.g., poly-A, GGX, GA). Replication slippage would result in length variation among the ensemble repeats within a gene, as has been observed in *MaSp1, MaSp2*, and *Flag* [26], [41], [49]. Because previously described *MaSp1* and *MaSp2* gene or cDNA fragments are substantially incomplete (typically<2000 bp) and represent the least homogenized parts of the genes (5′ or 3′ ends), it is unknown if these genes are composed of higher level aggregates of ensemble repeats. Thus, it remains to be seen whether the extreme repetitiveness and homogenization of *L. hesperus MaSp1* compared to *L. hesperus MaSp2* is a general feature of spider dragline fibroins, or whether this pattern is peculiar to black widows.

### Relationship to other silk proteins

Attempts to reconstruct evolutionary relationships among members of the spider silk gene family have relied exclusively on the non-repetitive C-terminus [11]–[12], [14], [26], [40], [69], but the N-terminus has great promise for phylogenetic reconstruction [38]–[39]. In our analyses, there was extensive congruence between trees based on N- and C-termini of silk gene family members (Figure 4C). A curious relationship found in both the N- and C-terminal phylogenetic trees is the grouping of *Latrodectus* major ampullate silk genes rather than a clade of *MaSp1* from all species separate from a *MaSp2* clade (Figure 4C). A similar sister relationship between *MaSp1* and *MaSp2* C-termini has been found for other species [10], [12], [40]. Given the striking conservation of repetitive amino acid motifs for each fibroin across divergent species, it seems unlikely that this pattern could result from independent duplication and convergence events. To explain the similarities in the repetitive regions by these means requires the convergence of thousands of nucleotides within a silk gene to encode either entirely MaSp1 or MaSp2 motifs, and for such convergences to have occurred multiple times in different spider lineages. Instead, recombination, selection, or the interaction of these two processes more likely explains the similarity of MaSp1 and MaSp2 N- and C-termini within species. Intergenic pairing during meiosis could be facilitated by the stretches of DNA coding for similar amino acid motifs, such as poly-A and GGX, in both *MaSp1* and *MaSp2*. For example, pairwise comparisons of the *L. hesperus MaSp1* and *MaSp2* genes show that they contain multiple regions of significant similarity spanning at least 100 bp (Figure 6). If recombination occurs between these two genes, it is less frequent than speciation events; *MaSp1* of *L. geometricus* and *L. hesperus* were clustered in the N-terminal trees and the C-terminal ML tree. Thus far, no single gene has been described that contains repeat units typical of both genes, which would provide the most convincing evidence for intergenic recombination. We did not find any clones in the *L. hesperus* genomic library that were positive for both *MaSp1* and *MaSp2,* nor did Sponner et al. [15] find double positive clones in a *Nephila clavipes* genomic library. However, there could be strong selection against proteins with a mixture of repeat units, while terminal recombinants may be tolerated. Convergent evolution could alternatively explain the grouping of *MaSp1* and *MaSp2* paralogs by their N- and C-termini. Selection could drive convergence of terminal amino acid sequences within species if similarity in these regions is necessary for accurate assembly of the two fibroins into a single fiber. Both proteins are exposed to the same environments, such as salt and pH gradients in the silk gland and duct [70], which could also favor evolutionary convergence of terminal domains.

### Non-coding sequence

Non-coding sequences upstream of major ampullate silk genes from different genera were too divergent to reliably align or identify regulatory elements other than the conserved motif CACG and the TATA-box identified by Motriuk-Smith et al. [38]. Although phylogenetic footprinting is a powerful tool for identifying novel regulatory elements, the appropriate level of divergence among species is critical [51]. The genera examined here, *Latrodectus, Nephila*, and *Argiope*, belong to three different families that shared a common ancestor ∼135–160 million years ago (MYA) [71]–[72]. In contrast, some of the most successful examples of phylogenetic footprinting involve more recent divergences (e.g. *Drosophila* spp. [73], *Saccharomyces* spp. [74]–[75], grasses [76], and primates [77]). Comparisons of human and rodent genomes, thought to have split ∼100 MYA [78], yield many novel regulatory elements, while extending divergence to mammals and birds (∼310 MYA [79]–[80]) causes a precipitous drop off in the ability to detect motifs [81]–[82]. In plants, the limit of motif detection is gene specific but appears to be reached when comparing poplars and *Arabidopsis* [83], which diverged ∼110 MYA [84]–[85]. Thus, given the divergence times of the spider taxa examined here, the fact that the promoter regions of their major ampullate silk genes retain any significant sequence similarity is notable.

In *Latrodectus*, ∼300 bp of upstream sequence could be reliably aligned. However, the ∼150 bp directly upstream of the start codon are more conserved than the adjacent, upstream non-coding sequence or synonymous sites in coding regions of the genes (Figure 5). We found a conserved motif in this region that matches the binding site for the Achaete-Scute family of transcription factors, which regulate neurogenesis and sensory mother cell development in *Drosophila* [86]–[87]. A homolog of this transcription factor family, called SGSF, shows a silk gland-restricted pattern of expression in *L. hesperus*, specifically to the tubuliform and major ampullate silk glands [88]. These are the only glands that appear to express *MaSp1* and *MaSp2* in appreciable quantities [40]. Experimental manipulation is needed to elucidate if SGSF or a related protein is, in fact, involved with regulating major ampullate silk gene expression in black widows and other spider species.

The conserved, upstream non-coding regions and the 3′ UTRs of *L. hesperus MaSp1* and *L. hesperus MaSp2* show evidence for stronger selective constraints than do *L. hesperus MaSp1* and *L. geometricus MaSp1* (lower K/K_s_, Figure 5). Although regulatory element evolution in the 3′ UTR has received less attention than in promoter regions, many genes display significantly conserved sequence motifs in the 3′ UTR [e.g. 89]–[91]. Additionally, experimental evidence has shown that elements in the 3′ UTR bind factors involved in posttranscriptional regulation [e.g. 92]–[93]. A striking example of 3′ regulation is in *Drosophila's Enhancer of split* Complex, which belongs to the same class of genes (beta helix-loop-helix) as *achaete* and *scute* [89], [94]–[96]. Taken together, our findings suggest selection on non-coding sequences for co-regulation of the paralogous dragline silk genes, *MaSp1* and *MaSp2*.

### Recipe for a high-performance biomaterial

The production of synthetic spider dragline silk is a major goal of biomimetics research [1], [29]. Though promising advances have been made with a variety of transgene constructs and host organisms, an exact mimic of a native dragline silk fiber has yet to be produced [e.g. 30], [33]–[35]. While artificial spinning is certainly an important consideration, a significant challenge to the efforts to create synthetic silk proteins has been the incomplete knowledge of spider silk genes. Thus far, all transgene constructs for recombinant silk proteins have relied on partial cDNA sequences from two orb-weaving species, *Nephila clavipes* and *Araneus diadematus* [e.g. 29]–[30], [32]–[35], [37], [97]. These truncated cDNAs encode only a fraction (typically 20% or less) of the repetitive region and the C-terminal domain. Experiments on recombinant silks made with and without the C-terminal region showed that the C-terminus was required for fibroins to form aggregates. Protein aggregation is an essential step in the precipitation of liquid spinning dope into a solid silk fiber [37], [98]. The C-terminus is not only necessary for aggregation of recombinant fibroins, but also for the formation of the characteristic crystalline structures that impart strength to dragline silk fibers [35]. As has been proposed for the C-terminus [37], the evolutionary conservation of the N-terminus suggests that this region is also functionally significant. For example, N-termini may play a central role in the proper transport of fibroins from secretory cells to silk gland lumen, aid in fiber formation, and contribute to the structural properties of silk fibers. In both *L. hesperus* MaSp1 and MaSp2, the N-terminal domain contains the most hydrophobic region of the entire fibroin (Figure 3). The next most hydrophobic region is the C-terminus. Sponner et al. [37] hypothesized that the hydrophobicity of the C-terminus was a key characteristic for its role in fibroin aggregation. The hydrophobic N-terminal region could thus similarly enhance silk fiber formation and mechanical properties. Another evolutionarily conserved aspect of spider fibroins is their extremely large size, which is also a feature of independently evolved insect fibroins. Thus, large size has been repeatedly selected for in the evolution of fibroin genes. Therefore, a complete silk gene, with full representation of the N- and C-terminal regions, the intervening repetitive sequence, and the transitions among these domains, should dramatically improve recombinant silk performance.

The complete gene sequences described here highlight the extraordinary molecular characteristics of spider silks. Black widow major ampullate silk genes are highly modular, exhibiting a hierarchical organization of iterated short motifs and ensemble repeats (groups of motifs). By characterizing full-length *MaSp1* and *MaSp2* genes, we were able to detect even higher-level repeats (aggregates of ensemble repeats) and uncover a striking difference in the degree of repeat homogenization between *MaSp1* and *MaSp2*. The extreme modularity of *MaSp1* (Figure 1) may reflect selection on the MaSp1 fibroin for perfect repeats, perhaps important for rapid and consistent spinning of high quality silk fibers. Sequence homogenization, however, is also due to molecular mechanisms, such as unequal crossing over (e.g., two large tandem repeats in Figure 2), and the interaction between selection and concerted evolution is a subject for further investigation. We have additionally identified putative regulatory elements that may enhance expression of transgenic silks. Thus, the clones sequenced here provide the complete genetic blueprints for the primary protein components of the major ampullate silk fiber. These designs hold critical information for the mass production of artificial fibers that accurately mimic the spectacular high-performance properties of native spider silk.

## Methods

### Genomic Library Construction and Screening

We targeted black widow silk genes because in addition to the exemplary properties of their silk, *Latrodectus hesperus* has one of the smallest known genome sizes for a spider (C-value of 1.29 picograms [99]), meaning that fewer genomic clones must be screened to find a gene of interest. Individuals were collected from a single locality in Riverside, California (USA), live frozen in liquid nitrogen, and stored at −80°C. High-molecular-weight DNA was isolated from the cephalothoraxes of eight individuals using a modified method of Sambrook and Russell [100]. Following isolation, DNA was mechanically sheared through a pipette tip and subsequently treated with End-Repair Enzyme Mix (Epicentre) to produce blunt 5′ phosphorylated ends. Fragments ranging from 38–50 kilobases were gel excised, purified, and ligated into pCC1FOS™ vector (Epicentre). Resulting fosmids were packaged using MaxPlax™ Lambda Packaging Extracts and transfected into Epi300-T1R *E. coli* cells following protocols for the CopyControl™ Fosmid Library Production kit (Epicentre). Approximately 100,000 recombinant *E. coli* colonies were picked and arrayed into 276 culture plates each containing 384 wells using a QPIX robotic picker (Genetix). Each culture plate was replicated and original stock plates containing 7.5% glycerol were stored at −80°C.

To efficiently screen the genomic library, fosmid DNA was extracted from cell cultures combined from a single 384-well plate, and such extractions were done for every plate in the library. Polymerase chain reaction (PCR) experiments targeting genes of interest were used to identify which plate contained one or more positive clones. Once the plate was identified, that plate was replicated twice, and cell cultures from the rows were combined to form 16 templates, while cell cultures from the columns were combined to form 24 templates. Templates were then PCR screened to identify individual clones containing the gene of interest. Primers targeting *MaSp1* and *MaSp2* were designed from *L. hesperus* cDNA clones [40] (*MaSp1*–N-terminal clone, EF595247; *MaSp2*–C-terminal clone, AY953075). The primers, LhMaSp1NF254, 5′-TGGCTTTCGCATCATCTGTAGC-3′ and LhMaSp1NR607, 5′-CTCCTTGACCATAACTAACTGGCTG-3′ amplified a 350 bp portion of the *MaSp1* 5′ region. Primers LhMaSp2_1086F, 5′-CATCAGCAGCAGGACCAAGTG-3′, and LhMaSp2_1337R, 5′-GCGTTGTCGGTGAAGATAAAGC-3′, amplified a 250 bp portion of the *MaSp2* 3′ region.

### Sequencing

Seven *MaSp1*-positive clones and three *MaSp2*-positive clones were found after screening half of the library. One positive clone for each gene was shotgun sequenced and assembled by Qiagen (Hilden, Germany) to 6× coverage for the *MaSp2*-positive clone and 8× coverage for the *MaSp1*-positive clone. This resulted in three contiguous sequences (contigs) for the *MaSp2*-positive clone with two gaps within the coding sequence and one directly after the stop codon. The 707 bp gap between the stop codon and the downstream contig was closed by sequencing directly off the fosmid clone using primers designed from the C-terminal coding region of *MaSp2* and for the beginning of the downstream contig (all primer sequences used in this study are available upon request). Primer walking to close the two gaps within the *MaSp2* coding sequence was not possible due to its repetitive nature. Instead the clone was digested with NotI and BamHI (New England Biolabs) and a 9 kb restriction fragment containing almost the entire repetitive portion of *MaSp2* was subcloned into pZErO™-2 plasmids (Invitrogen) and electroporated into Epi-400 *E. coli* (Epicentre). The subclone was partially digested with PstI (New England Biolabs) and 2000-3000 bp fragments were gel excised and ligated into PstI digested and dephosphorylated pZErO™-2. Ligation products were electroporated into TOP10 *E. coli* (Invitrogen). A library of 96 PstI partial-digest clones were arrayed and sequenced in one direction. Sequences were assembled independently and using the fosmid contigs as a backbone in SEQUENCHER v4.5 (Gene Codes Corp.), requiring 100% identity for high-quality bases. Ten clones spanned the first gap (111 bp) and 18 clones spanned the second gap (632 bp) with no less than 5× sequence coverage of any base along the length of the NotI-BamHI subclone. No disagreement between the sequences of the subclone and the fosmid contigs was found.

Shotgun sequencing of a *MaSp1*-postive clone resulted in a single contig containing the entire coding sequence of *MaSp1* and the vector. However, this contig was ∼7000 bp smaller than expected based on restriction digests. This missing sequence was determined by PCR amplifying with AccuPrime™ Taq DNA Polymerase High Fidelity (Invitrogen) and primers designed from both ends of the contig. The 7890 bp PCR product was sequenced with at least 2× coverage by primer walking. Additionally, the fosmid was directly sequenced at intervals along the gap to ensure that no mutations had been introduced by the PCR amplifcation. Experimental restriction digests of the *MaSp1*-positive and *MaSp2*-positive clones matched predicted restriction sites in the final sequences, verifying that assembly had not erroneously excluded repetitive sequence.

### Sequence analysis

Nucleotide sequences were conceptually translated using the standard genetic code. Base composition, amino acid content, codon usage, and Kyte and Doolittle [50] hydrophilicity predictions were calculated in MacVector™ (Oxford Molecular Group). Amino acid sequences were considered to start at the first methionine in frame. The first M on the MaSp1 sequence corresponded to the conserved start position identified by Rising et al. [39] (see also Figure 4A). The MaSp2 sequence also displayed an M at this position, but the first in frame M codon was 9 bp upstream (Figure 4A). Pairwise K, K_s_, and K_n_ were calculated using DnaSp v4.0 [101] excluding gaps and missing data.

Predicted amino acid sequences of all currently published N-termini were aligned (Figure 4A), making corrections to the nucleotide sequences of *L. geometricus MaSp1, A. bruennichi CySp2*, and *N. clavipes Flag* according to the modifications described in Rising et al. [39]. Alignments of N- and C-terminal amino acid sequences were made separately using default parameters in ClustalW (MacVector™). The C-terminal alignment was modified slightly such that the first position of the C-terminal Flag sequences aligned with the first position of the other sequences (Figure 4B). Amino acid alignments were used to guide nucleotide alignments, which formed the basis for phylogenetic analyses. Heuristic ML and MP searches were performed in PAUP* [102] using TBR (tree bisection reconnection) branch swapping and 10,000 (MP) or 100 (ML) random stepwise addition replicates. Support for clades was evaluated with 1000 (MP) or 100 (ML) bootstrap pseudoreplicates (of all characters), and 100 (MP) or 1 (ML) random stepwise addition replicates per pseudoreplicate. ML analyses treated gaps as missing data. MP analyses were performed treating gaps as missing data and as a 5^th^ state. Optimal model parameters for ML analyses were calculated with MODELTEST [103]. The N-termini fit the HKY+G [104] model of evolution (transitions/transversions = 1.24; gamma = 0.9058). The C-termini fit the TrN+G [105] model of evolution (A<>G = 2.34; C<>T = 1.27; transversions = 1; gamma = 1.34). To further evaluate tree structure and clade support in a model-based framework, Bayesian analyses were carried out using MRBAYES v.3.1.2 [106]. The same model of evolution determined by MODELTEST was used but parameter values were evaluated during the Bayesian analysis. Default priors and Metropolis-coupled, Markov-chain, Monte Carlo (MCMC) sampling procedures were executed for two independent runs, sampled every 100^th^ generation, carried out simultaneously. Convergence was assessed every 1000^th^ generation and the posterior distribution was considered adequately sampled when the standard deviation of split frequencies of these two runs dropped below 0.01 (<1 million generations). A second analysis was run for 10 million generations (sampling every 500) to ensure that a longer sampling time did not change the results. For each run, the first 50% of sampled trees were discarded as burnin prior to calculating the majority rule consensus tree.

Comparisons of genes with MultiPipMaker were done using the “high sensitivity low time limit” option. Each major ampullate silk gene with upstream sequence was sequentially input as the reference to obtain maximal pairwise alignments. AVID alignments were made using default parameters and viewed on the VISTA browser <www-gsd.lbl.gov/vista/> [107]–[108]. Global alignments of conserved non-coding sequence identified by MultiPipMaker were made using default parameters in ClustalW and modified manually. Approximately 300 bp of upstream sequence were scanned against insect transcription factor binding sites in the TRANSFAC 6.0 database using the program PATCH™ v1.0 [57] with a minimum match of 6 and a maximum mismatch of 2.

Open reading frames on the black widow genomic clones were identified using the ORFFinder program on the NCBI website <http://www.ncbi.nlm.nih.gov/gorf/gorf.html>, with a minimum cutoff of 300 nucleotides.
